# Harnessing artificial intelligence to address substance use disorders in critically ill adolescents: a synergistic approach

**DOI:** 10.1186/s13613-025-01553-w

**Published:** 2025-09-03

**Authors:** Zekai Yu

**Affiliations:** School of Computer Science and Technology, Hangzhou Dianzi University, No. 1158, No. 2 Road, Xiasha Higher Education Zone, Hangzhou, 310018 Zhejiang Province China

We read with great interest the recent study by Markus et al. (2025) investigating the impact of substance use disorders (SUDs) on critical care outcomes in adolescents with sepsis. The authors demonstrated that SUDs significantly increased organ dysfunction and resource utilization without affecting mortality—a finding with profound implications for clinical practice [1]. While the study highlights the urgent need for early identification and intervention in this vulnerable population, we propose that integrating artificial intelligence (AI) into care pathways could amplify these efforts and address existing limitations.

## AI-Driven early screening and risk stratification

The study underscores the underdiagnosis of SUDs in adolescents due to clinician time constraints and inconsistent screening. AI-powered tools, trained on multimodal data (e.g., EHR narratives, social determinants of health (SDOH), and behavioral patterns), could automate SUD risk stratification. For instance, natural language processing (NLP) algorithms analyzing clinical notes for terms like “non-prescribed opioid use” or “cannabis dependence” could flag high-risk patients missed by structured diagnostic codes. A 2017 study by Acion et al. demonstrated that machine learning models achieved AUC values ranging from 0.793 to 0.820 in SUD detection, significantly outperforming traditional coding methods [2].

## Predictive analytics for personalized care

The authors observed that SUDs exacerbated organ dysfunction but did not affect mortality, suggesting complex interactions between substance use and sepsis pathophysiology. AI could unravel these dynamics by integrating real-time biomarkers (e.g., cytokine profiles, microbial data) with longitudinal substance use histories [3]. Predictive models, such as those analyzed by Wang et al. (2025) where hand-crafted features improved accuracy, could pinpoint SUD patients at highest risk for multiorgan failure, informing precision resuscitation protocols despite external validation challenges [4]. Furthermore, reinforcement learning algorithms could optimize vasopressor dosing or ventilator settings in SUD patients, whose altered pharmacokinetics may demand precision adjustments.

## Ethical considerations and implementation challenges

While AI offers transformative potential, its integration requires caution. Algorithmic transparency, patient consent for data use, and clinician training are paramount. Collaborative efforts between critical care societies and AI developers, as advocated by Sisk et al. (2024), could establish guidelines for ethical AI deployment in pediatric settings [5].

## Conclusion

Markus et al. have illuminated a critical gap in adolescent sepsis management. By coupling their findings with AI technologies, we may advance from retrospective risk identification to proactive, personalized care. Future research should prioritize validating AI models in multicenter cohorts and exploring their impact on long-term outcomes, such as substance relapse prevention. Together, clinical expertise and AI innovation could redefine care paradigms for this high-risk population.

## Data Availability

Not applicable.
